# Measuring energy, macro and micronutrient intake in UK children and adolescents: a comparison of validated dietary assessment tools

**DOI:** 10.1186/s40795-019-0312-9

**Published:** 2019-11-21

**Authors:** Linda A. Bush, Jayne Hutchinson, Jozef Hooson, Marisol Warthon-Medina, Neil Hancock, Katharine Greathead, Bethany Knowles, Elisa J. Vargas-Garcia, Lauren E. Gibson, Barrie Margetts, Sian Robinson, Andy Ness, Nisreen A. Alwan, Petra A. Wark, Mark Roe, Paul Finglas, Toni Steer, Polly Page, Laura Johnson, Katharine Roberts, Birdem Amoutzopoulos, Darren C. Greenwood, Janet E. Cade

**Affiliations:** 1 Nutritional Epidemiology Group, School of Food Science and Nutrition, University of Leeds, LS2, 9JT, Leeds, UK; 2 Quadram Institute Bioscience, Norwich, NR4 7UA UK; 3 EuroFIR AISBL, 40 Rue Washington, 1050 Brussels, Belgium; 4 School of Primary Care and Population Sciences, Faculty of Medicine, University of Southampton, Southampton General Hospital, Southampton, SO16 6YD UK; 5 NIHR Newcastle Biomedical Research Centre, Newcastle upon Tyne Hospitals NHS Foundation Trust and Newcastle University, Newcastle upon Tyne, UK; 6 National Institute of Health (NIHR) Bristol Biomedical Research Centre, Nutrition Theme, University, Hospitals Bristol NHS Foundation Trust and the University of Bristol, Bristol, BS2 8AE UK; 7 NIHR Southampton Biomedical Research Centre, University of Southampton and University Hospital, Southampton NHS Foundation Trust, Southampton, UK; 8 Centre for Innovative Research Across the Life Course (CIRAL), Faculty of Health and Life Sciences, Coventry University, Coventry, CV1 5FB UK; 9 Global eHealth Unit, Department of Primary Care and Public Health, Imperial College London, London, SW7 2AZ UK; 10 AGE Research Group, Newcastle University, Newcastle, UK; 11 MRC Elsie Widdowson Laboratory, Cambridge, CB1 9NL UK; 12 Centre for Exercise, Nutrition and Health Sciences, School for Policy Studies, University of Bristol, Bristol, BS8 1TH UK; 13 Public Health Section, School of Health and Related Research (ScHARR), University of Sheffield, S10 2TN, Sheffield, UK; 14 Public Health England, London, SE1 8UG UK; 15 Faculty of Medicine and Health, Division of Biostatistics, University of Leeds, Leeds, LS2 9JT UK

**Keywords:** Dietary assessment, Macronutrients, Micronutrients, Validation, Mean difference, limits of agreement

## Abstract

**Background:**

Measuring dietary intake in children and adolescents can be challenging due to misreporting, difficulties in establishing portion size and reliance on recording dietary data via proxy reporters. The aim of this review was to present results from a recent systematic review of reviews reporting and comparing validated dietary assessment tools used in younger populations in the UK.

**Methods:**

Validation data for dietary assessment tools used in younger populations (≤18 years) were extracted and summarised using results from a systematic review of reviews of validated dietary assessment tools. Mean differences and Bland-Altman limits of agreement (LOA) between the test and reference tool were extracted or calculated and compared for energy, macronutrients and micronutrients.

**Results:**

Seventeen studies which reported validation of 14 dietary assessment tools (DATs) were identified with relevant nutrition information. The most commonly validated nutrients were energy, carbohydrate, protein, fat, calcium, iron, folate and vitamin C. There were no validated DATs reporting assessment of zinc, iodine or selenium intake. The most frequently used reference method was the weighed food diary, followed by doubly labelled water and 24 h recall. Summary plots were created to facilitate comparison between tools. On average, the test tools reported higher mean intakes than the reference methods with some studies consistently reporting wide LOA. Out of the 14 DATs, absolute values for LOA and mean difference were obtained for 11 DATs for EI. From the 24 validation results assessing EI, 16 (67%) reported higher mean intakes than the reference. Of the seven (29%) validation studies using doubly labelled water (DLW) as the reference, results for the test DATs were not substantially better or worse than those using other reference measures. Further information on the studies from this review is available on the www.nutritools.org website.

**Conclusions:**

Validated dietary assessment tools for use with children and adolescents in the UK have been identified and compared. Whilst tools are generally validated for macronutrient intakes, micronutrients are poorly evaluated. Validation studies that include estimates of zinc, selenium, dietary fibre, sugars and sodium are needed.

## Background

According to the Health Survey for England, 30% of UK children aged 2–15 are classified as overweight or obese [1]. Underweight also occurs, particularly in children from lower socio-economic backgrounds at around 5% [2]. In addition, the National Diet and Nutrition Survey (NDNS, 2016) identified low intakes of some micronutrients, particularly iron, selenium, calcium and zinc, and high intakes of non-milk extrinsic sugars amongst children and adolescents in the UK [3].

Accurate measurement of dietary intake in children and adolescents is important to capture dietary patterns, eating behaviours and to monitor diet quality. No consensus exists regarding the best methodology for collecting dietary / food intake data from younger populations since dietary assessment tools (DATs) often consist of modified tools previously developed for adults [4]. Although children aged 6–11 years tend to be more enthusiastic and willing compared to adolescents when reporting food intakes [5], children younger than 8 years old can face further challenges related to their reading and cognitive skills, particularly when DATs require more advanced cognitive skills or the reporting period is longer than a few days [6]. Therefore parental/adult assistance is required to obtain dietary information on meal frequency, portion sizes and energy intake for younger children [4, 6].

Food habits become less structured as children get older and more independent; as adolescents they are more selective around their food choices and consumption of meals outside the home increases [7]. Exposure to an ‘obesogenic environment’ is associated with an increase in overweight and obesity amongst adolescents in the UK [7, 8]. The increasing use of new technologies such as mobile food records and wearable devices, where sensors detect physical eating patterns, has helped to address some limitations in traditional dietary methodologies [9, 10]. These methods are likely to be more appealing than paper based records to younger generations [11].

Valid and reliable dietary assessment methods are crucial to track changes in children’s and adolescent’s diets, and to estimate the nutritional adequacy of nutrient intake. Ideally a DAT should be validated in a representative sample of the population in which it will be used [12]. Previous reviews have addressed the validity of DATs in school-aged or pre-school children and discussed the challenges that still remain to improve the quality of dietary information obtained from children and adolescents [4, 5, 13, 14]. Most reviews have focussed on specific aspects of diet, such as fruits and vegetables or energy [15, 16]; or have only included tools used in specific types of study, for example intervention studies [6]. None of the existing reviews provided results in a format allowing comparison between tools based on limits of agreement between the test and reference tool. A systematic review of reviews [17], including details of tools validated on infants, children and adolescents has been undertaken by the DIETary Assessment Tool NETwork (Diet@NET) partnership project and made available on the www.nutritools.org website to enable researchers to compare and choose the DAT most suitable for their research purpose [18].

In this paper, we quantify the extent of the validity of a range of dietary assessment tools for children and adolescents, and identify gaps in the tools available. Individual tools and nutrients generated from the validation studies identified in our recent systematic review are compared [17]. We focus on comparing the results of nutrient validations of DATs used in children and adolescents in the UK, where absolute intakes have been evaluated.

## Methods

A detailed description of the methods has been published elsewhere [17], but briefly consisted of a systematic review of reviews of validated DATs. A search strategy was undertaken in 11 online databases to identify validated DATs in UK populations. Reviews that had conducted validation analysis of DATs using nutrient biomarkers or self-reported methods to measure energy, macro or micronutrient intake were retrieved and later screened by title and abstract to evaluate their eligibility for inclusion.

The inclusion and exclusion criteria applied for both the reviews and the identified DATs are in Table 1 and also published elsewhere [17]. All reviews meeting the inclusion criteria were independently assessed by two reviewers; papers in the relevant reviews which reported tools used in a child or adolescent population (≤18 years) and had validation results on this population are reported in more detail here. Papers reporting on the individual tools and validations were then obtained. Data extracted from these were the administration method of the DAT (person reporting: self, by proxy, interviewer), nutrient database, timeframe covered by the tool, its comparator (reference method), the nutrients validated, age range, demographics, sample size, gender, statistical methods used and findings.

**Table 1:** Inclusion and exclusion criteria applied to the reviews and DATs

Reviews		DATs	
Inclusion criteria	Exclusion criteria	Inclusion criteria	Exclusion criteria
• Reviews that validated a DAT against a biomarker or another self-reported tool against energy, macro or micro nutrients or food groups • Reviews published since 1st January 2000	• Reviews that exclusively evaluated tools assessing inadequacy of diets in terms of malnutrition • Commentaries, editorials or other opinion articles	• Tools measured in a UK population • Be able to measure dietary intake • Validation results can be entered on thenutritools website	• DATs measuring eating disorders, food preferences, feeding practices or inadequacy of diets • Lifestyle based tools (e.g. diet plus physical activity) • DATS measuring the purchasing of foods / drinks • Tools that assessed specific dietary interventions (e.g. Atkins, Mediterranean diet) • Non-UK tools

### Statistical analysis

Results of studies validating energy and/or nutrients that reported the mean difference (MD) and the Bland-Altman limits of agreement LOA, or had sufficient information to calculate them, were included in the data analysis and associated figures. For each validation study, mean differences in estimated nutrient intake and the upper and lower Bland Altman LOA between the tested DAT and reference method were extracted (mean tool – mean reference method) or calculated from means and standard deviations (SD) of the mean difference if provided (LOAs = mean diference ±1.96 SD (or 2 SDs in some cases)). LOAs were also estimated for studies that did not report the SD of the mean difference, but reported the mean estimated intake for the tool and reference method and SD of the means. The mean difference provides useful information on the direction and level of bias [6] between the DAT and reference method, whilst the LOA provides information about how precise estimates are by indicating how well the two methods agree for an individual. These results are presented in summary plots produced using Stata version 14.1. Validation results reporting different genders and age groups are displayed individually.

The arrows on the plots represent the upper and lower LOA, with the central dot of each line representing the mean difference (MD) between the two methods (The DAT name and author are displayed on the left and the reference method type, validation author, lifestage and sample size of the validation population is displayed on the right for each validation result). The circles around the mean represent studies that have a sample size of ≥50, with larger circles representing larger sample sizes. Mean values to the left of the zero on the x-axis represent lower mean intakes and those on the right of the zero represent higher mean intakes reported by the test DAT compared to the reference. Wider LOA arrows represent more variation of the MD between the DAT and reference method within the sample; therefore narrower LOA indicate better relative validity. So wider LOA indicate a noisier tool, with greater opportunity for disagreement for an individual. The best way to use the plot is to define a priori the limits of maximum acceptable differences i.e. the limits of agreement expected.

## Results

**Fig. 1:**
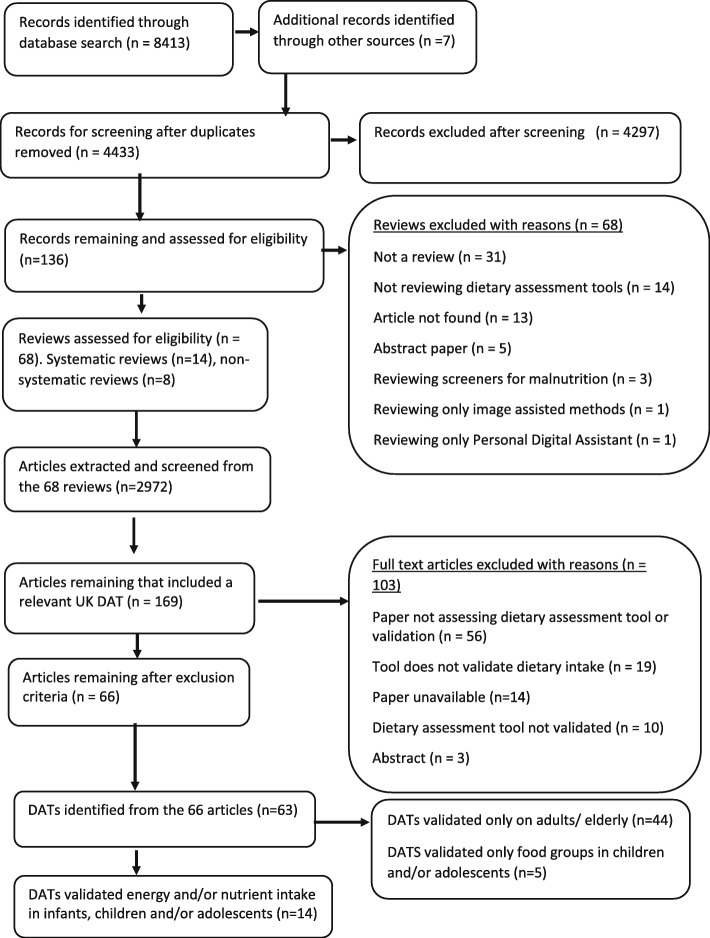
PRISMA flow chart showing number of articles included at each phase and number of dietary assessment tools (DATS) found

The number of reviews and individual papers identified from the on-line database search from the systematic review of reviews [17] is shown in Fig. 1 and the search algorithm can be found in appendix 1. Further additional records were identified through reference tracking and internet searches. After removing duplicates and screening the title and abstract 136 articles remained. Screening of these 136 articles resulted in 68 reviews including 2972 articles. Of these, 169 articles included a UK based DAT. Following exclusion of articles not fitting our crtieria (Table 1), 66 articles remained containing 63 validated DATs of which 19 were DATs that separately reported results for infant, children and adolescent populations [17]. 14 DATs assessed energy, macro and/or micronutrient intake in infants, children and adolescents and the LOA validations of these from 14 publications are reported in this paper (Table 2 and detailed in Table 3). Five DATs that focussed solely on food group intake in this population were excluded from this paper [36–40]. The remaining DATs exclusively analysed dietary intake in adult and elderly populations and the validation of these are reported elsewhere.

**Table 2:** summary of the number of dietary assessment tools, validation study publications and validation studies from the systematic review of reviews

	Number of dietary assessment tools (DATs)	Number of validation study publications^a^	Number of validation studies^b^
Total from systematic review of reviews	63	66	89
Results for adults	49c, d	49	71
Results for infants, children and adolescents (IC&A)	19c, e	19	22
Total for IC&A validating nutrients	14	14	17
Total for IC&A with limits of agreement (LOA) plotted	11	11^f^	14

^a^More than one DAT may have been validated in a published validation study, and some DATs may have more than one validation study publication

^b^This takes into account more than one DAT validated in a publication i.e. each DAT validation is counted as a validation study

^c^5 tools were assessed on both adults and Infants, children or adolescents

^d^5 tools assessed on adults focused on foods only

^e^5 tools assessed on IC&A focused on foods only

^f^data was extracted from these 11 publications to produce the energy summary plot showing 24 validations by gender and age/lifestage

**Table 3:** General characteristics of UK dietary assessment tools and their validation studies in children and adolescents

Test Dietary Assessment Tool				Validation Studies						
First author and year	Administration method	Nutrient database	First author and year	Macronutrients validated	Micronutrients validated	Food groups included (Y/N)	Life stage, age range Cohort (M/F)	Time span		Statistical Method Used
								Test DAT	Reference method	
Weighed food diary										
Davies [19] (1994)	By adult proxy	MCW4	Davies [19] (1994)	E	0	N	Children & Infants (1.5–4.5 yr) *81 (42/39)*	4d consecutive	10d (DLW)	Mean Difference (relative bias); CC (NR); LOA
Livingstone [20] (1992)	Self (12–18 yr)); By adult proxy (7 + yr)	MCW4 inc. supplementary food composition data	Livingstone [20] (1992)	E	0	N	Children & Adolescents (7–18 yr) *58 (29/29)*	7d consecutive	10 – 14d (DLW)	Mean Difference LOA
Estimated food diary										
Lanigan [21] (2001)	By adult proxy	COMP-EAT v.5	Lanigan [21] (2001)	E, PRO, FAT, CHO	0	N	Infants (6–24 months) *DLW – 21* *Weighed Food Diary – 72*	5d	7d (DLW) & 5d (Food Diary)	Mean Difference LOA
Semi-weighed food diary										
Holmes [22] (2008)	Self (12+ yr) By adult proxy (< 5 yr), adult proxy / child combined (6-11 yr) Interview	MCW5	Holmes [22] (2008)	E, PRO, FAT, CHO, DF	RET, Vit B1, B9, C, Ca, Fe	N	Children (2–10) & adolescents (11-17 yr) 124 (70/52)	4d	4d (weighed food diary)	Mean Difference; LOA^**c**^
Dietary recall										
Carter [23] (myfood24) (2015)	Self; Interview	MCW7	Albar [24] (2016)	^a^All assessed	Sodium	Y	Adolescents *75 (37/38)*	2d (non-consecutive)	2d (non-consecutive 24-h recall)	Mean Difference; CC (ICC); Cross Classification LOA; Weighted Cohen’s kappa
^b^Foster [25] (INTAKE24) (2013)	Self	MCW	Bradley ^[^ [11] (2016)	E, PRO, FAT,CHO, NSP, SUG,	Vit C, calcium, iron	Y	Adolescents	4d (Results reported data on participants completing any number of days)	4d recall (Results reported data on participants completing any number of days)	Mean ratios; LOA (ratio)^b^
Holmes [22] (2008)	Self (12+ yr) By adult proxy (< 5 yr), adult proxy / child combined (6-11 yr) Interview	MCW5	Holmes [22] (2008)	E, PRO, FAT, CHO, DF	RET, Vit B1, B9, C, Ca, Fe	N	Children (2–10_& adolescents (11-17 yr) 124 (70/52	4d	4d (weighed food diary)	Mean Difference; LOA^**c**^
Johnson [26] (1996)	Interview	Food Intake Analysis	Reilly [27] (2001)	E	0	N	Children (3–4 yr) *41 (23/18)*	3d (MPR)	7d (DLW)	Mean Difference; LOA
			Montgomery [28] (2005)	E	0	N	Children (4.5–7 yr) *63 (32/31)*	3d (Inc. 1 weekend d)	2d (DLW)	Mean Difference (bias); LOA
			Johnson [26] (1996)	E	0	N	Children (4–7 yr) (12/12)	3d (MPR)	14d (DLW)	Mean Difference; LOA
Food frequency questionnaire										
McKeown [29] (EPIC FFQ) (2001)	Self	MCW	Lietz [30] (2002)	E, PRO, FAT, CHO,	Ca, K, Na	N	Adolescents (11.8–13.2 yr) *50 (32/18)*	1d	7d (Food diary)	Mean Difference; CC (S); Cross Classification; LOA
^b^Robinson [31] (2007)	By adult proxy	MCW5	Marriot [32] (2008)	E, PRO, FAT, CHO, SUG	^a^All assessed	N	Infants (6 months) *50 (25/25)*	1d	4d (weighed food diaries)	Mean Difference (%); CC(S); LOA^b^
^b^Robinson [31] (2007)	By adult proxy	MCW5	Marriot [33] (2009)	E, PRO, FAT, CHO, SUG	^a^All assessed	N	Infants (12 months) *50 (27/23)*	1d	4d (weighed food diaries)	Mean Difference (%); CC (S); LOA^b^
Food checklist										
Cade [34] (CADET) (2006)	Combination of Self and adult proxy (parent, school dinner supervisor)	DANTE	Cade [34] (2006)	^a^All assessed	Ca, Fe, B9, K, Vit C	Y	Children (3–7 yr) *180 (100/80)*	1d	1d (weighed food diary)	Mean Difference; CC (S); LOA
			Christian [35] (2015)	E, PRO, CHO, FAT,SUG, DF	Na, Ca, Vit C	Y	Children (8–11 yr) *67 (33/34)*	1d	1d (weighed food diary)	Mean Difference; CC (P) LOA
Holmes [22] (2008)	Self (12+ yr) By adult proxy (< 5 yr), adult proxy / child combined (6-11 yr) Interview	MCW5	Holmes [22] (2008)	E, PRO, FAT, CHO, DF	RET, Vit B1, B9, C, Ca, Fe	N	Children (2–10) & adolescents (11-17 yr) 124 (70/52)	4d	4d (weighed food diary)	Mean Difference; LOA^**b**^
Diet history										
Livingstone [20] (1992)	Self (12–18 yr)); By adult proxy (7 + yr)	MCW4	Livingstone [20] (1992)	E	0	N	Children & Adolescents (3–18 yr) *78 (41/37)*	1d	10-14d (DLW)	Mean Difference; LOA

^a^All assessed = Macronutrients: E (Energy), PRO (Protein), UR (Urinary Nutrogen), CHO, (Carbohydrate) FAT, DF (Dietary Fibre / NSP),; MUFA (Monounsaturated Fatty Acids), PUFA (Polyunsaturated Fatty Acids), SFA (Saturated Fatty Acids), SUG (Sugar). *Ca* Calcium, *Na* Sodium, *Fe* Iron, *K* Potassium, *RET* Retinol

^b^Results expressed as a ratio or percentage so not shown on the summary plots

^**c**^LOA calculated from information reported

MCW = McCance & Widdowson; DLW (Doubly Labelled Water); CC (Correlation coefficient), S (Spearman), P (Pearson); ICC(Intra-class correlation coefficient); LOA (Limits of Agreement)

### Characteristics of the reviews

The age range for infant, children and adolescent populations covered by the reviews varied with some focussing on a specific age group such as ≤5 years [41], ≤ 7 years [42], 3–9 years [43], or ≤ 11 years [44], or adolescents [45, 46], with some including specific variables such as pregnant teenagers [47], or children with cerebral palsy [48]. Reviews that focussed exclusively on food groups were not included in this review.

### Characteristics of the DATs

The characteristics of the 14 DATs which assessed energy, macro- and/or micronutrients are displayed in Table 3. Three of the tools (21%) were a modified version of a tool previously developed for children [26, 42] or adults [29]. The most frequently used tool was the 24-h recall (*n* = 4, 29%) followed by the food frequency questionnaire (FFQ) (*n* = 3, 21%), food checklist (*n* = 2, 14%), weighed food diary (n = 2, 14%), with the semi-weighed food diary, estimated food diary and diet history having one tool each for inclusion. All studies assessed energy intake (EI) with 10 (71%) assessing protein, 10 (71%) fat, 10 (71%) carbohydrate and 10 (71%) of the DATs validating at least three macronutrients. The most common micronutrient assessed was calcium (*n* = 8, 57%) followed by iron and vitamin C (both *n* = 7, 50%) with three (21%) reporting folate intakes. There were no validated DATs reporting assessment of zinc, iodine or selenium intake in either children or adolescents. Out of the 14 DATs, three (21%) also included food groups in their analysis.

A range of validated DATs had been used across different age ranges. For example, in infants ≤3 years three studies used food diaries [19, 21, 22], one a 24-h recall [26], and two FFQ’s were used that covered different age ranges [30]. In children 3–11 years, tools used were food diaries [19, 20, 22], dietary recall [22, 26], food checklists [22, 33] and diet history [20]. For adolescents aged 12–18 years, methods used were again food diaries [20, 22], 24-h recalls [22, 24, 25], FFQ [29], food checklist [22] and diet history [20]. The majority of studies validated one DAT in their analysis, with one study that used three different DATs [22] and another study that used two different DATS [20].

All DATs included in this review specified which food database they used with McCance and Widdowsons ‘The Composition of Foods’ (MCW) food tables or a database based upon MCW being the main nutrient database used by the DATs (*n* = 11, 79%).

### Characteristics of the validation studies

Most of the validation studies had a sample size of ≤50. Results for mean nutrient intakes for the test DATs were generally greater than the reference method for all nutrients, indicating a reporting of higher mean intakes by the test DAT compared to the reference. A total of 17 validation studies (ie. more than one DAT could be validated in a publication) from 14 papers were identified for the 14 DATs which included LOA or information to calculate them (LOAs of the three DATs developed by Holmes et al. were calculated from reported information [22]) (Table 2). Two validation studies that reported the LOA as a ratio [11] or as a percentage [33] instead of absolute values could not be included in the summary plots or table of validation results. In total three comparator (reference) methods were used for validation with five (31%) being doubly labelled water (DLW), two (13%) dietary recalls and nine (56%) food diaries. One study used two different validation methods which were DLW and weighed food diary [21].

**Fig. 2:**
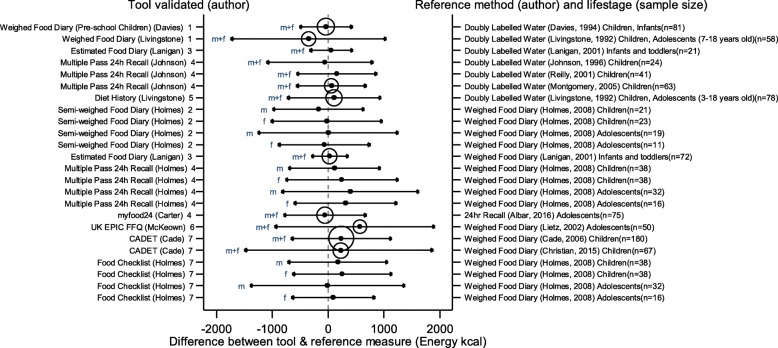
Summary plot for studies validating energy intake between tool and reference method in infants, children and adolescents. m = males; f = females; m + f = males & females. Relative sample size circle produced where *n* > 50. 1 = food diary weighed, 2 = food diary semi-weighed, 3 = food diary estimated, 4 = dietary recall, 5 = diet history, 6 = FFQ, 7 = food check list

**Fig. 3:**
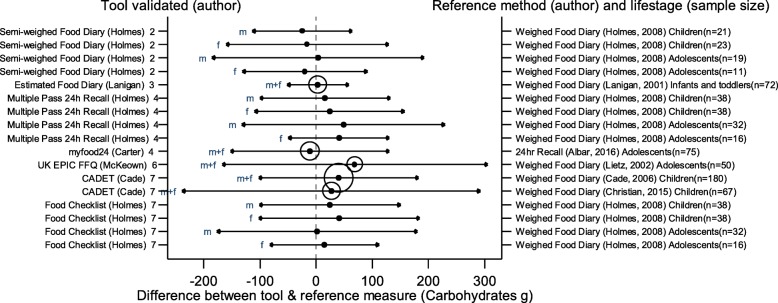
Summary plot for studies validating carbohydrate intake between tool and reference method in infants, children and adolescents. m = males; f = females; m + f = males & females. Relative sample size circle produced where *n* > 50. 1 = food diary weighed, 2 = food diary semi-weighed, 3 = food diary estimated, 4 = dietary recall, 5 = diet history, 6 = FFQ, 7 = food check list

**Fig. 4:**
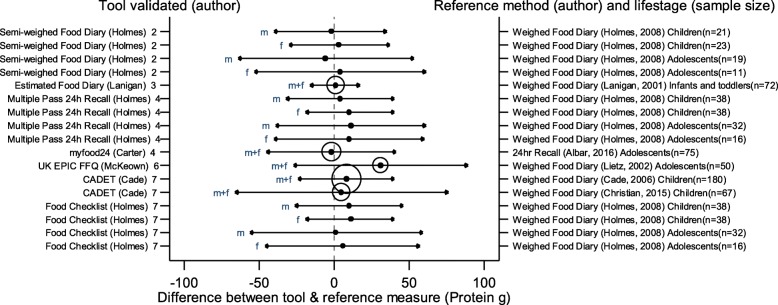
Summary plot for studies validating protein intake between tool and reference method in infants, children and adolescents. m = males; f = females; m + f = males & females. Relative sample size circle produced where *n* > 50. 1 = food diary weighed, 2 = food diary semi-weighed, 3 = food diary estimated, 4 = dietary recall, 5 = diet history, 6 = FFQ, 7 = food check list

**Fig. 5:**
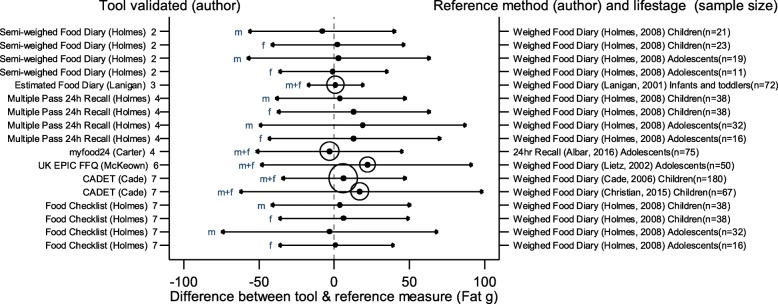
Summary plot for studies validating fat intake between tool and reference method in infants, children and adolescents. m = males; f = females; m + f = males & females. Relative sample size circle produced where *n* > 50. 1 = food diary weighed, 2 = food diary semi-weighed, 3 = food diary estimated, 4 = dietary recall, 5 = diet history, 6 = FFQ, 7 = food check list

**Fig. 6:**
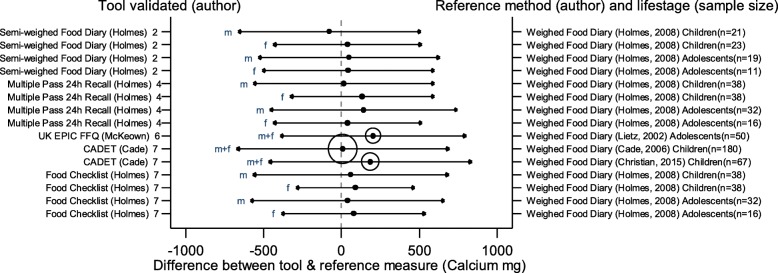
Summary plot for studies validating calcium intake between tool and reference method in infants, children and adolescents. m = males; f = females; m + f = males & females. Relative sample size circle produced where *n* > 50. 1 = food diary weighed, 2 = food diary semi-weighed, 3 = food diary estimated, 4 = dietary recall, 5 = diet history, 6 = FFQ, 7 = food check list

**Fig. 7:**
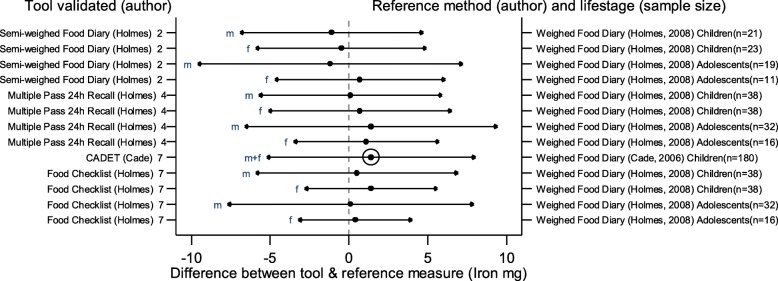
Summary plot for studies validating iron intake between tool and reference method in infants, children and adolescents. m = males; f = females; m + f = males & females. Relative sample size circle produced where *n* > 50. 1 = food diary weighed, 2 = food diary semi-weighed, 3 = food diary estimated, 4 = dietary recall, 5 = diet history, 6 = FFQ, 7 = food check list

**Fig. 8:**
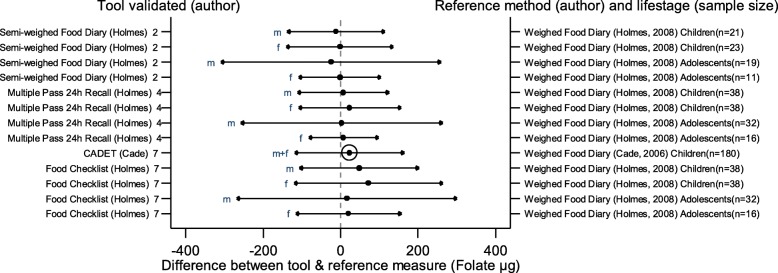
Summary plot for studies validating folate intake between tool and reference method in infants, children and adolescents. m = males; f = females; m + f = males & females. Relative sample size circle produced where *n* > 50. 1 = food diary weighed, 2 = food diary semi-weighed, 3 = food diary estimated, 4 = dietary recall, 5 = diet history, 6 = FFQ, 7 = food check list

**Fig. 9:**
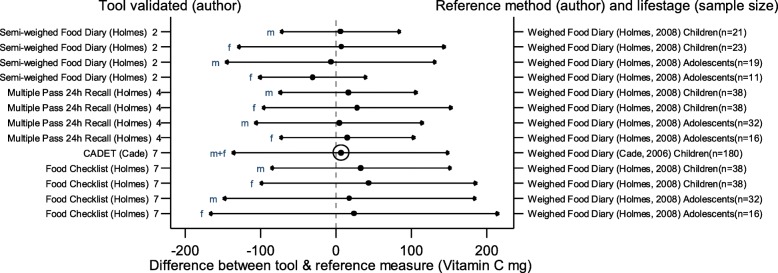
Summary plot for studies validating vitamin C intake between tool and reference method in infants, children and adolescents. m = males; f = females; m + f = males & females. Relative sample size circle produced where *n* > 50.1 = food diary weighed, 2 = food diary semi-weighed, 3 = food diary estimated, 4 = dietary recall, 5 = diet history, 6 = FFQ, 7 = food check list

The statistical methods used to assess the difference between the test DATs and the reference methods for nutrients and energy varied, with one validation study (6%) using five methods [26], (mean difference [MD], cross classification, LOA, correlation coefficient and weighted Cohens kappa) and one study (6%) using four methods [30]. On average 2.4 statistical methods were used by the validation studies in this review. Figures 2 to 9 show the summary plots of the nutrient intakes between the test DAT and reference method with a table in appendix 2 providing the actual numerical values for the mean difference (MD) and LOA between the test DAT and reference.

Participants in the validation studies were recruited from a range of institutions such as playgroups [20], schools [11, 34], GP Practices [21], personal addresses [19, 22], newspaper articles [26], existing studies [30, 33] and email / posters [24]. Studies were conducted in different areas across England, and one study took place in Belfast [20]. No studies were carried out in Wales or Scotland.

### Energy and macronutrients

Out of the 14 DATs, absolute values for LOA and mean difference had been obtained for 11 DATs for EI which were compared in summary plots. Figures 2, 3, 4 and 5 show the summary plot results for energy and three macronutrients (carbohydrates, protein and total fat). From the 24 validation results reported by gender and age group assessing EI, 16 (67%) reported higher mean intakes than the reference. Of the seven (29%) validation studies using doubly labelled water (DLW) as the reference, results for the test DATs were not substantially better or worse than those using other reference measures. The limits of agreement tended to be wide, at around half of the daily requirements for macronutrients, with even wider limits in relation to requirements for micronutrients. There were no clear differences between mean difference and LOA for studies evaluating tools for children or adolescents, although there was a tendency for the LOA to be narrower for studies of children than for adolescents. Davies et al. weighed food diary [19] validation on infants and children (aged 1.5–4.5 years old) and the Lanigan et al. estimated food diary [21] validated on infants (aged 6–24 months) had a low mean difference and relatively narrow LOA (MD 33 kcal, LOA − 229 to 364 kcal and MD 57 kcal, LOA − 331 to 445 kcal respectively); whilst the results of Livingstone et al. weighed food diary [20] (across 7–18 year age range) showed a poorer agreement (MD -351 kcal, LOA − 1747 to 1045 kcal). The narrowest LOA for energy for adolescents was reported in the myfood24 validation (MD -55 kcal, LOA − 797 to 687 kcal); however this online recall tool was compared to a similar self-reported method, a paper 24 h recall.

Seven DATs had validation results for CHO, protein and fat intake. From the 17 validation results reported for these, most showed higher intakes with the test DAT than the reference, with the majority (*n* = 16, 94%) using the weighed food diary as the reference method. The Holmes et al. semi-weighed food diary tended to under-report intake compared to the weighed diary [22]. For these macronutrients, the narrowest difference in the means and LOA was found in the Lanigan et al. estimated food diary validations on 6–24 month olds [21], MD 3 g, LOA − 51 to 58 g (CHO) MD 1 g, LOA − 16 to 17 g (protein) and MD 1, LOA − 18 to 20 g (fat). The McKeown et al. FFQ [29] validated on young adolescents (11–13 years old) represented the greatest mean difference and one of the widest LOAs, MD 574 kcal, LOA − 956 to 1912 (EI), MD 69, LOA − 167 to 305 (CHO), MD 31, LOA − 27 to 89 (protein) and MD 22, LOA − 49 to 92 (fat) [30]. The Christian et al. validation of the CADET tool [35] on children aged 8–11 years also had wide LOA (MD = 228, LOA − 1497 to 1881 (EI), MD = 27, LOA − 238 to 292 (CHO), MD = 5, LOA − 66 to 79 (protein) and MD = 17, LOA − 63 to 99 (fat). However, the earlier validation of CADET [34] on younger children, 3–7 year olds, which had the largest sample size (180) of all the validations, had similar MD but much narrower LOA (MD = 237, LOA − 665 to 1139 (EI), MD = 40, LOA − 102 to 182 (CHO), MD = 8, LOA − 24 to 40 (protein) and MD = 6, LOA − 35 to 48 (fat). Summary plots for dietary fibre and total sugars are not reported here because of very limited results for these nutrients (see Table 3).

In general, DATs that tested a semi-weighed or estimated food diary to validate against another weighed food diary displayed the lowest difference in the means, compared with other tools. Also, DATs using infants and children for validations showed closer results between the DAT and reference compared to validations using adolescents.

### Micronutrients

Figures 6, 7, 8 and 9 display the summary plots for four micronutrients (calcium, iron, folate and vitamin C). Only four tools were validated on all four micronutrients: three tools reported by Holmes et al. [22], plus CADET reported by Cade et al. [34], and only CADET had a sample size over 50. All validation studies for micronutrients used the weighed food diary as the reference method. LOAs tended to be wider for males, especially adolescent males. Most of the 15 validation results reported by gender and age group for calcium intake, and the 13 validation results assessing iron, folate and vitamin C, reported higher mean intakes in the test DAT than the reference method (number of studies with DAT higher than reference for calcium =14 (93%), iron = 10, (77%), folate = 9, (69%), vitamin C = 11, (85%)). Of the three tools reported by Holmes et al. [22], the Food Check List had the greatest mean differences and/or the widest LOAs for children aged 2–10 for folate and vitamin C. Holmes et al. semi-weighed tool tended to yield lower intakes [22]. Otherwise there was no clear best overall method. Results for sodium were limited so a summary plot was not generated for analysis.

## Discussion

The systematic review of reviews [17] identified 14 DATs validated on UK infants, children and adolescents which assessed energy, macro and/or micronutrient intake. This was considerably fewer than the number of DATs validated on adults (*n* = 44) assessing nutrients, partly due to a smaller number of DATs being available for children and adolescents to use. Not all macro- and micronutrients were validated for these 14 DATs. No validations for the nutrients zinc, iodine or selenium intakes were reported. These nutrients have been identified as insufficient in some UK children and adolescent populations [49] and low intakes are associated with negative health outcomes [50–52]. It is therefore important to obtain reliable intakes of these nutrients. Also only a small number of validation results were reported for total sugar (*n* = 3), dietary fibre (*n* = 5) and sodium (n = 5); reliable assessment of sugar intakes is important because reduction of sugar intake is a priority with current intakes exceeding recommendations in the UK [49].

This report focuses on comparing Bland-Altman limits of agreement (LOA) generated from studies validating DATs in children and adolescents. This approach measures agreement and systematic bias between a tool and comparator [53], unlike the commonly used correlation coefficient. The majority of these validated DATs showed similar, though slightly higher, mean intakes compared to the reference method. Estimated intakes also differed depending on the tool type and reference method used as demonstrated by the wide range of LOA. Additionally, the width of the LOA between two dietary assessment methods may be affected by sample sizes, with validation sample sizes of ≥50 enabling greater accuracy when estimating particular nutrients [54]. The smallest bias (MD) and narrowest LOA for macronutrients assessed were found in studies with some of the largest samples sizes (e.g. Lanigan et al. [21] and Davies et al. [19] with sample sizes of 72 and 81 respectively). Furthermore, these studies were on infants and young children (up to age 4.5 years old), where dietary intake was completed by adult carers which may increase accuracy. A wide LOA was found for the Livingstone weighed food diary validated against the DLW (*n* = 58, 38). This may be due to the the wide age range (7–18 years old) with older children more involved in recording intake, and/or because data for this study was obtained via different sources such as parents, child minders and school lunch supervisory staff some of whom may not have been trained adequately in completing the DAT [20]. Shared responsibility for reporting food intake between different adult carers can compromise accuracy [6]. In addition, variability in adolescent self-reported dietary intake has been shown to be much higher than for younger children or adults [13].

The majority of DATs used a self-reported reference method and therefore reported only relative validity; this has limitations since the same type of errors can occur in both the tool being validated and the reference and therefore they are not strictly independent of each other [54]. This will result in little relative bias, because they both suffer from the same bias of self-report. This would explain why DATs that tested a semi-weighed or estimated food diary against another weighed food diary had the lowest difference in the means, compared with other tools. Although biomarkers such as urinary nitrogen or the DLW method are objective measures, without correlated sources of error, they are challenging to use with young children and are expensive. DLW measures total energy expenditure (TEE) using respiratory eqs [20]. and is considered the ‘gold standard’ for measuring free living TEE but relies on a consistent CO_2_ production [55]. Also, dietary intake and DLW TEE are not always assessed over similar time frames [6], which may be problematic for validating long-term dietary measures.

Adolescent females in particular may be more likely to under-report their energy intake due to issues with body weight and image [5]; therefore it is important to report validation studies by gender. However, some validation results in this review did not sub-divide results for males and females; none of the validation studies using DLW reported them separately. The majority of DATs that assessed EI amongst adolescents using other reference methods did subdivide males and females, but there were no singificant differences in the mean intake between the DAT and reference methods between males and females. However LOAs for males were usually wider.

Food diaries were used both as a test DAT and a reference method, with estimated or semi-weighed methods sometimes being used for the test DAT and weighed food diaries often used as the reference method. Weighed food diaries, in particular, can be more rigorous in assessing the accuracy of dietary intake in children and adolescents than other self/proxy-reported methods because it attempts to assess current rather than past dietary intakes and parents are able to weigh foods and subsequently establish more accurate portion sizes. However, limitations can still occur with this method due to social desirability bias from parent-completers and older self-completers, as well as the burden of self-reporting, particularly amongst those with low literacy levels [5]. Estimated food diaries using standard household units of measurement (e.g. cups, spoons) and / or photographs or food models can reduce some of this burden but can have increased risk of misreporting [56].

Four of the validated DATs were recalls which are beneficial for evaluating dietary intake in children and adolescents because they do not require good literacy skills if administered by interviewer, have a low respondent burden [5] and are straightforward to administer [22]. However, this method has particular limitations such as recall bias and over-reporting [6] as well as under-reporting [27] for particular healthy or less healthy food types respectively. Although adults normally help to obtain dietary intake for children ≤8 years [4, 6], misreporting can occur if they are not fully aware of food consumed or are unable to quantify portion sizes [4, 6]. Some of these issues can be reduced when a combination of words and pictures to are used to report dietary intake [24, 35] .

Three validated DATs were FFQs; this type of tool generally has low cost and low participant burden [16, 57]. Despite these advantages FFQs do not allow recording of individual ingredients of meals, affecting accuracy of assessment [29]. Also, overestimation and misreporting is a common feature with an FFQ [6]. The UK EPIC FFQ tool validated on adolescents showed the greatest overestimation of EI, macronutrient and calcium intake between the DAT and reference method which was a weighed food diary [30]. Overestimation of nutrient intakes may be more likely for tools if they use adult portion sizes [4], a feature of the McKeown FFQ tool. Furthermore, recognition that adolescents are less motivated and cooperative with recording dietary intake may be a limitation that can lead to inconsistencies in results [5].

One diet history tool was validated [20], which may have a lower probability of misreporting than some other methods [6]. Two validated DATs were food checklists; this may be effective in younger populations due to their ease of use when recording dietary intake [22]. However, many checklists do not account for quantity or portion size making nutrient analysis difficult. The development of alternative tools such as the CADET [34] which includes mean children’s portion sizes from the National Diet and Nutrition Survey, supports more robust nutrient analysis.

The application of technology for dietary assessment methods may be more appealing for children and adolescents because they are confident with tablet and smartphone use which can therefore increase compliance. Additionally, such tools may assist children and adolescents with lower cognitive and literacy skills to report their food intake. However, challenges remain relating to following procedures associated with these DATs, food databases and portion size estimation [58]. In this review, two DATs were identified which made use of new technologies which were both on-line 24-h recalls. These tools, which were INTAKE24 [25] and myfood24 [23, 24], both include instructions for ease of use as well as features such as colour photographs to help with portion size estimation. The EI validation results of myfood24 showed one of the smallest mean differences and narrowest LOA; however this was validated using a similar tool, a paper-based 24 h recall [24]. A more recent publication has found that the myfood24 online 24-h recall is comparable to the more time-consuming and costly interviewer-based 24-h recall across a range of biomarker measures [59]. A review of new technology-based dietary assessment tools has identified limitations with these approaches and provided guidance for reporting studies [58].

The concerns surrounding the quality of reporting in nutritional epidemiology and research can make recommending one DAT over another difficult. In recognition of this, new guidelines have been developed by the STROBE-nut consortium [60]. Information and data collected here has been put on the www.nutritools.org website allowing researchers to review and compare both UK and international DATs, identify their strengths and weaknesses and compare LOA validation results in summary plots, allowing researchers to select the most appropriate tools for their research question. Functions will allow creation of web-based tools using the food questionnaire creator, ensuring easier data collection and nutrient analysis, improving the options available for researchers. The website also hosts the recently developed expert consensus Best Practice Guidelines (BPGs), providing support to researchers when looking to select a suitable DAT [18]. These can be accessed through the www.nutritools.org website.

### Study strengths and limitations

The inclusion and presentation of the MD and LOA in summary plots provides easier comparisons between the test DAT and validation method. LOA is preferable to most other comparison methods aiming to assess population mean intakes, as it measures agreement as well as systematic bias between a tool and comparator [53]. Whereas the use of the correlation coefficient, despite being commonly used in dietary assessment, is limited, showing strength and direction of the linear relationships between variables rather than agreement between methods [61]. Ideally, a number of statistical approaches should be used in dietary validation studies to provide more insight into the validity of a particular DAT [61]. A limitation of our analyses is that the LOA were not reported or could not be calculated for all validation studies identified. Additionally, nutrient intakes were evaluated at an absolute level, however ideally these should be energy adjusted to partially correct for dietary misreporting, and this should be encouraged for future validations. The use of relative validity from self/proxy-reported reference measures, as opposed to absolute validity using biomarkers, for the majority of the test DATs may have resulted in measurement error; as a result of both test and reference measures being self-reported leading to closer agreements between the tools than if independent biomarkers had been used. Results presented here are limited to the information provided in the validation study reports, and whilst we report type of tool, reference method and lifestage there may be other unreported biases present.

The comprehensive search strategy ensured the systematic review process was thorough. However, identification of all DATs validated on children and adolescents in UK populations could not be guaranteed. Despite the date restriction on the published reviews (≥ January2000) there was no date restriction on the actual DAT included for analysis raising the question of whether tools developed over 25–30 years ago are still fit for purpose today. Not all UK countries were represented by the studies in this review with the majority (*n* = 13) being in England.

### Recommendations

From this review it appears that few dietary assessment tools are fit for purpose, the LOA indicate poor relative validity fior most DATs. We recommend use of more objectively measured tools (reducing systematic components of measurement error), and tools designed for easy repeat administration (reducing the random component of measurement error). More DATs should be developed and existing DATs updated to ensure validity for a wider range of dietary constituents. Few studies presented data on nutrient densities, which have been shown to be slightly less prone to misreporting. Few studies consistently presented validation for ranking of individuals, which can be useful in establishing risk factors for disease, whilst public health recommendations require target intakes rather than target ranks. However, the biggest weakness in the validation studies was lack of an objective reference, such as recovery biomarkers. We recommend that future validation studies include information on all these aspects to provide a more complete picture of the appropriateness of their dietary assessment tool.

There is a potential to use new mobile and online technologies, especially for adolescents, with tools validated using independent biomarkers where available, to assess nutrient intakes, this data is missing for zinc, iodine, selenium and limited for sugar intake in children and adolescents. Sugar intakes exceed recommendations in the UK [3], and is associated with poor nutritional status in children [32, 62, 63]; making it an area of current public concern which has resulted in a UK soft drinks levy. Studies also need to incorporate a range of more appropriate statistical methods, such as the Bland-Altman LOA, to ensure reliability and comparability of results. The issue of underreporting in adolescent females still requires further research, particularly with DLW as the reference method, and validations for males and females should be reported separately.

## Conclusions

This review has identified validated DATs that assessed energy, macro and micronutrients in children and adolescents in the UK. Summary plots have been created to facilitate comparison between tools. Whilst most tools were validated for macronutrient intakes, micronutrients had inadequate evaluation. Some nutrients, such as zinc, iodine and selenium did not have any validation studies reported; whilst studies assessing sugar, fibre and sodium intakes were limited. Valid DATs are needed to support monitoring of nutritional status in children and adolescents.

## Supplementary information

**Additional file 1.** systematic review of reviews search algorithm. This is an example of a search run in Ovid MEDLINE(R). The search was initially conducted in May/June 2015, then updated in October 2016, and was restricted to reviews published between January 2000 and October 2016. (DOCX 12 kb)

**Additional file 2.** UK validation study results for dietary assessment tools by nutrient in children/adolescents (0 to 18 years). This table provides the numerical values from the published validation studies of dietary assessment tools in children/adolescents which are included in the summary plots. (DOCX 29 kb)

## Data Availability

The data analysed during for this paper is shown in appendix 2, and this information can also be found under each validation study on the Nutritools website www.nutritools.org. This data has been extracted from each original published article.

## Abbreviations

DAT: Dietary assessment tool
Diet@NET: DIETary Assessment Tool NETwork
DLW: Doubly labelled water
EI: Energy intake
FFQ: Food frequency questionnaire
LOA: Limits of agreement
MCW: McCance and Widdowsons ‘The Composition of Foods’ food Tables
MD: Mean difference
SD: Standard deviation
TEE: Total energy expenditure
