# Supplementary material for: Measuring energy, macro and micronutrient intake in UK children and adolescents: a comparison of validated dietary assessment tools
Source: BMC Nutr. 2019 Nov 21;5:53. doi: 10.1186/s40795-019-0312-9 (PMC7050749; doi:10.1186/s40795-019-0312-9)
Supplement: Supplementary file 1 — Additional file 1. systematic review of reviews search algorithm. This is an example of a search run in Ovid MEDLINE(R). The search was initially conducted in May/June 2015, then updated in October 2016, and was restricted to reviews published between January 2000 and October 2016. (DOCX 12 kb) [file 40795_2019_312_MOESM1_ESM.docx]

**Appendix 1**

1     exp diet/

2     Nutritional status.mp.

3     diet* adj2 intake*.mp

4    diet* adj2 qualit*.mp.

5     food adj2 intake*.mp.

6     nutri* adj2 intake*.mp.

7     diet* adj2 habit*.mp.

8 food adj2 habit.mp.

9     diet* pattern* or meal pattern*.mp.

10   food group*.mp.

11   nutrient*.mp.

12   macro-nutrient* or macronutrient.mp.

13   micro-nutrient or micronutrient.mp.

14   energy intake*.mp.

15   1 or 2 or 3 or 4 or 5 or 6 or 7 or 8 or 9 or 10 or 11 or 12 or 13 or 14

16   diet* adj2 (method* or tool* or survey* or record* or assess*).mp.

17   diet* adj2 (recall* or questionnaire* or histor* or instrument*).mp.

18   nutrition* adj2 (survey* or assess* or instrument*).mp. (27252)

19   food adj2 (questionnaire* or record* or recall* or diar* or checklist* or screener*).mp

20   24* adj2 recall.mp.

21   multiple pass.mp

22   FFQ*.mp

23   diet* adj2 (measure* or analys*).mp

24   nutri* adj2 measur*.mp

25   16 or 17 or 18 or 19 or 20 or 21 or 22 or 23 or 24

26   valid*.mp.

27   reliab*.mp.

28   reproduc*.mp.

29   calibrat*.mp.

30 repeatab*.mp

31 feasib*.mp

32 evaluat*.mp

33   26 or 27 or 28 or 29 or 30 or 31

34   review*.mp

35   meta-analy*.mp.

36   search*.mp.

37   systematic* adj2 (approach or analys*).mp.

38   33 or 34 or 35 or 36

39     15 and 25 and 32 and 37
