# Supplementary material for: Measuring energy, macro and micronutrient intake in UK children and adolescents: a comparison of validated dietary assessment tools
Source: BMC Nutr. 2019 Nov 21;5:53. doi: 10.1186/s40795-019-0312-9 (PMC7050749; doi:10.1186/s40795-019-0312-9)
Supplement: Supplementary file 2 — Additional file 2. UK validation study results for dietary assessment tools by nutrient in children/adolescents (0 to 18 years). This table provides the numerical values from the published validation studies of dietary assessment tools in children/adolescents which are included in the summary plots. (DOCX 29 kb) [file 40795_2019_312_MOESM2_ESM.docx]

**Appendix 2** UK validation study results assessing validity of DATs in children / adolescents (0 to 18 years)

| **Validation author & year (& Tool author if different)** | **Reference method** | **Nutrient assessed & unit** | **No of participantss** | **Gender (age)** | **Dietary assessment tool (DAT)** | **Administration method** | **Mean difference (tool minus reference)** | **LOA** |
| --- | --- | --- | --- | --- | --- | --- | --- | --- |
| Lanigan *et al.* 2001 | DLW | Energy (Kcal) | 21 | M & F  (6 - 12M) | EFD | Parent | 57 | -331 to 445 |
| Davies et al.  1994 | DLW | Energy (Kcal) | 81 | M & F  (1.5 - 4.5y) | WFD | Parent | -37 | -513 to 439 |
| Livingstone et al.  1992 | DLW | Energy (Kcal) | 58 | M & F  (7 - 18y) | WFD | Parent (children < 9 y,  Children > 10 to 18y | -351 | -1747 to 1045 |
| Livingstone et al 1992 | DLW | Energy (Kcal) | 78 | M & F  (3 - 18y) | DH | Parent (children < 9 y,  Children > 10 to 18y | 108 | -734 to 951 |
| Reilly et al  2001  (Johnson) | DLW | Energy (Kcal) | 41 | M & F  (3 - 4y) | MPR | Interviewer | 158 | -564 to 879 |
| Montgomery et al 2005  (Johnson) | DLW | Energy (Kcal) | 63 | M & F  (4.5 - 7y) | MPR | Interviewer | 60 | -569 to 688 |
| Johnson et al 1996 | DLW | Energy (Kcal) | 24 | M & F  (4-7y) | MPR | Interiewer | -54 | -1102 to 807 |
| Lanigan *et al.* 2001 | WFD | Energy (Kcal) | 72 | M &F  (6 - 24m) | EFD | Parent | 33 | -299 to 364 |
| Holmes et al. 2008 | WFD | Energy (Kcal) | 21 | M  (2 - 10y) | S-WFD | Parent | -172 | -994 to 650 |
| Holmes et al. 2008 | WFD | Energy (Kcal) | 23 | F  (2 - 10y) | S-WFD | Parent | -25 | -1025 to 975 |
| Holmes et al. 2008 | WFD | Energy (Kcal) | 19 | M  (11 -17y) | S-WFD | Self | 1 | -1259 to 1261 |
| Holmes et al. 2008 | WFD | Energy (Kcal) | 11 | F  (11 -17y) | S-WFD | Self | -69 | -895 to 757 |
| Holmes et al. 2008 | WFD | Energy (Kcal) | 38 | M  (2 - 10y) | MPR | Parent | 116 | -710 to 942 |
| Holmes et al. 2008 | WFD | Energy (Kcal) | 38 | F  (2 - 10y) | MPR | Parent | 248 | -762 to 1258 |
| Holmes et al. 2008 | WFD | Energy (Kcal) | 32 | M  (11 - 17y) | MPR | Self | 396 | -836 to 1628 |
| Holmes et al (2008) | WFD | Energy (Kcal) | 16 | F  (11-17yr) | MPR | Self | 312 | -612 to 1236 |
| Holmes et al. 2008 | WFD | Energy (Kcal) | 38 | M  (2 - 10y) | Food checklist | Parent | 173 | -723 to 1069 |
| Holmes et al. 2008 | WFD | Energy (Kcal) | 38 | F  (2-10y) | Food checklist | Parent | 255 | -637 to 1147 |
| Holmes et al. 2008 | WFD | Energy (Kcal) | 32 | M  (11 - 17y) | Food checklist | Self | -11 | -1399 to 1377 |
| Holmes et al. 2008 | WFD | Energy (Kcal) | 16 | F  (11 - 17y) | Food checklist | Self | 89 | -655 to 843 |
| Cade et al. 2006 | WFD | Energy (Kcal) | 180 | M&F  3 - 7y) | Food checklist | Parent/Interviewer/ self | 237 | -665 to 1139 |
| Christian et al. 2015  (Cade) | WFD | Energy (Kcal) | 67 | M&F  (8 - 11y) | Food checklist | Parent/Interviewer/ self | 228 | -1497 to 1881 |
| Lietz et al. 2002  (McKeown) | WFD | Energy (Kcal) | 50 | M&F  (11-13y) | FFQ | Self | 574 | -956 to 1912 |
| Lanigan *et al.* 2001 | WFD | Protein (g) | 72 | M&F  (6-24m) | EFD | Parent | 1 | -16 to 17 |
| Holmes et al. 2008 | WFD | Protein (g) | 21 | M  (2 - 10y) | S-WFD | Parent | -2 | -40 to 35 |
| Holmes et al. 2008 | WFD | Protein (g) | 23 | F  (2 - 10y) | S-WFD | Parent | 3 | -30 to 37 |
| Holmes et al. 2008 | WFD | Protein (g) | 19 | M  (11 -17y) | S-WFD | Self | -6 | -64 to 53 |
| Holmes et al. 2008 | WFD | Protein (g) | 11 | F  (11 -17y) | S-WFD | Self | 4 | -53 to 61 |
| Holmes et al. 2008 | WFD | Protein (g) | 38 | M  (2 - 10y) | MPR | Parent | 4 | -32 to 40 |
| Holmes et al. 2008 | WFD | Protein (g) | 38 | F  (2 - 10y) | MPR | Parent | 10 | -19 to 40 |
| Holmes et al. 2008 | WFD | Protein (g) | 32 | M  (11-17y) | MPR | Self | 11 | -39 to 61 |
| Holmes et al (2008) | WFD | Protein (g) | 16 | F  (11-17yr) | MPR | Self | 10 | -40 to 60 |
| Holmes et al. 2008 | WFD | Protein (g) | 38 | M  (2-10y) | Food checklist | Parent | 10 | -26 to 46 |
| Holmes et al. 2008 | WFD | Protein (g) | 38 | F  (2-10y) | Food checklist | Parent | 11 | -19 to 40 |
| Holmes et al. 2008 | WFD | Protein (g) | 32 | M  (11-17y) | Food checklist | Self | 1 | -56 to 59 |
| Holmes et al. 2008 | WFD | Protein (g) | 16 | F  (11-17y) | Food checklist | Self | 6 | -46 to 57 |
| Cade et al. 2006 | WFD | Protein (g) | 180 | M&F  (3-7y) | Food checklist | Parent/Interviewer/ self | 8 | -24 to 40 |
| Christian et al. 2015  (Cade) | WFD | Protein (g) | 67 | M&F  (8-11y) | Food checklist | Parent/Interviewer/ self | 5 | -66 to 76 |
| Lietz et al. 2002  (McKeown) | WFD | Protein (g) | 50 | M&F  (11-13y) | FFQ | Self | 31 | -27 to 89 |
| Lanigan *et al.* 2001 | WFD | CHO (g) | 72 | M&F  (6-24m) | EFD | Parent | 3 | -51 to 58 |
| Holmes et al. 2008 | WFD | CHO (g) | 21 | M  (2-10y) | S-WFD | Parent | -25 | -113 to 64 |
| Holmes et al. 2008 | WFD | CHO (g) | 23 | F  (2-10y) | S-WFD | Parent | -16 | -160 to 129 |
| Holmes et al. 2008 | WFD | CHO (g) | 19 | M  (11-17y) | S-WFD | Self | 4 | -185 to 192 |
| Holmes et al. 2008 | WFD | CHO (g) | 11 | F  (11-17y) | S-WFD | Self | -20 | -131 to 91 |
| Holmes et al. 2008 | WFD | CHO (g) | 38 | M  (2-10 y) | MPR | Parent | 16 | -100 to 132 |
| Holmes et al. 2008 | WFD | CHO (g) | 38 | F  (2-10y) | MPR | Parent | 24 | -109 to 157 |
| Holmes et al. 2008 | WFD | CHO (g) | 16 | F  (11-17y) | MPR | Self | 41 | -49 to 130 |
| Holmes et al. 2008 | WFD | CHO (g) | 32 | M  (11-17y) | MPR | Self | 49 | -132 to 229 |
| Holmes et al. 2008 | WFD | CHO (g) | 38 | M  (2-10y) | Food checklist | Parent | 24 | -101 to 150 |
| Holmes et al. 2008 | WFD | CHO (g) | 38 | F  (2-10y) | Food checklist | Parent | 41 | -102 to 184 |
| Holmes et al. 2008 | WFD | CHO (g) | 32 | M  (11-17y) | Food checklist | Self | 2 | -176 to 180 |
| Holmes et al. 2008 | WFD | CHO (g) | 16 | F  (11-17y) | Food checklist | Self | 15 | -82 to 112 |
| Cade et al. 2006 | WFD | CHO (g) | 180 | M&F  (3-7y) | Food checklist | Parent/Interviewer/ self | 40 | -102 to 182 |
| Christian et al. 2015  (Cade) | WFD | CHO (g) | 67 | M&F  (8-11y) | Food checklist | Parent/Interviewer/ self | 27 | -238 to 292 |
| Lietz et al. 2002  (McKeown) | WFD | CHO (g) | 50 | M&F  (11-13y) | FFQ | Self | 69 | -167 to 305 |
| Lanigan *et al.* 2001 | WFD | Fat (g) | 72 | M&F  (6-24m) | EFD | Parent | 1 | -18 to 20 |
| Holmes et al. 2008 | WFD | Fat (g) | 21 | M  (2-10y) | S-WFD | Parent | -8 | -57 to 41 |
| Holmes et al. 2008 | WFD | Fat (g) | 23 | F  (2-10y) | S-WFD | Parent | 2 | -42 to 47 |
| Holmes et al. 2008 | WFD | Fat (g) | 19 | M  (11-17y) | S-WFD | Self | 3 | -58 to 64 |
| Holmes et al. 2008 | WFD | Fat (g) | 11 | F  (11-17y) | S-WFD | Self | -1 | -37 to 36 |
| Holmes et al. 2008 | WFD | Fat (g) | 38 | M  (2-10y) | MPR | Parent | 4 | -39 to 48 |
| Holmes et al. 2008 | WFD | Fat (g) | 38 | F  (2-10y) | MPR | Parent | 13 | -38 to 64 |
| Holmes et al. 2008 | WFD | Fat (g) | 16 | F  (11-17y) | MPR | Self | 13 | -44 to 71 |
| Holmes et al. 2008 | WFD | Fat (g) | 32 | M  (11-17y) | MPR | Self | 19 | -50 to 88 |
| Holmes et al. 2008 | WFD | Fat (g) | 38 | M  (2-10y) | Food checklist | Parent | 4 | -42 to 51 |
| Holmes et al. 2008 | WFD | Fat (g) | 38 | F  (2-10y) | Food checklist | Parent | 6 | -37 to 50 |
| Holmes et al. 2008 | WFD | Fat (g) | 32 | M  (11-17y) | Food checklist | Self | -3 | -75 to 69 |
| Holmes et al. 2008 | WFD | Fat (g) | 16 | F  (11-17y) | Food checklist | Self | 1 | -37 to 40 |
| Cade et al. 2006 | WFD | Fat (g) | 180 | M&F  (3-7y) | Food checklist | Parent/Interviewer/ self | 6 | -35 to 48 |
| Christian et al. 2015  (Cade) | WFD | Fat (g) | 67 | M&F  (8-11y) | Food checklist | Parent/Interviewer/ self | 17 | -63 to 99 |
| Lietz et al. 2002 | WFD | Fat (g) | 50 | M&F  (11-13y) | FFQ | Self | 22 | -49 to 92 |
| Holmes et al. 2008 | WFD | Iron (mg) | 21 | M  (2-10y) | S-WFD | Parent | -1.1 | -6.9 to 4.7 |
| Holmes et al. 2008 | WFD | Iron (mg) | 23 | F  (2-10y) | S-WFD | Parent | -0.5 | -6 to 4.9 |
| Holmes et al. 2008 | WFD | Iron (mg) | 19 | M  (11-17y) | S-WFD | Self | -1.2 | -9.6 to 7.2 |
| Holmes et al. 2008 | WFD | Iron (mg) | 11 | F  (11-17y) | S-WFD | Self | 0.7 | -4.7 to 6.1 |
| Holmes et al. 2008 | WFD | Iron (mg) | 38 | M  (2-10y) | MPR | Parent | 0.1 | -5.7 to 5.9 |
| Holmes et al. 2008 | WFD | Iron (mg) | 38 | F  (2-10y) | MPR | Parent | 0.7 | -5.1 to 6.5 |
| Holmes et al. 2008 | WFD | Iron (mg) | 16 | F  (11-17y) | MPR | Self | 1.1 | -3.5 to 5.7 |
| Holmes et al. 2008 | WFD | Iron (mg) | 32 | M  (11-17y) | MPR | Self | 1.4 | -6.6 to 9.4 |
| Holmes et al. 2008 | WFD | Iron (mg) | 38 | M  (2-10y) | Food checklist | Parent | 0.5 | -5.9 to 6.9 |
| Holmes et al. 2008 | WFD | Iron (mg) | 38 | F  (2-10y) | Food checklist | Parent | 1.4 | -2.8 to 5.6 |
| Holmes et al. 2008 | WFD | Iron (mg) | 32 | M  (11-17y) | Food checklist | Self | 0.1 | -7.7 to 7.9 |
| Holmes et al. 2008 | WFD | Iron (mg) | 16 | F  (11-17y) | Food checklist | Self | 0.4 | -3.2 to 4.0 |
| Cade et al. 2006 | WFD | Iron (mg) | 180 | M&F  (3-7y) | Food checklist | Parent/Interviewer/ self | 1.4 | -5.2 to 8.0 |
| Holmes et al. 2008 | WFD | Folate µg) | 21 | M  (2-10y) | S-WFD | Parent | -12 | -138 to 114 |
| Holmes et al. 2008 | WFD | Folate (µg) | 23 | F  (2-10y) | S-WFD | Parent | -2 | -140 to 136 |
| Holmes et al. 2008 | WFD | Folate (µg) | 19 | M  (11-17y) | WFD | Self | -25 | -309 to 259 |
| Holmes et al. 2008 | WFD | Folate (µg) | 11 | F  (11-17y) | WFD | Self | -2 | -108 to 104 |
| Holmes et al. 2008 | WFD | Folate (µg) | 38 | M  (2-10y) | MPR | Parent | 7 | -111 to 125 |
| Holmes et al. 2008 | WFD | Folate (µg) | 38 | F  (2-10y) | MPR | Parent | 24 | -108 to 156 |
| Holmes et al. 2008 | WFD | Folate (µg) | 16 | F  (11-17y) | MPR | Self | 8 | -82 to 98 |
| Holmes et al. 2008 | WFD | Folate (µg) | 32 | M  (11-17y) | MPR | Self | 3 | -257 to 263 |
| Holmes et al. 2008 | WFD | Folate (µg) | 38 | M  (2-10y) | Food checklist | Parent | 48 | -106 to 202 |
| Holmes et al. 2008 | WFD | Folate (µg) | 38 | F  (2-10y) | Food checklist | Parent | 72 | -120 to 264 |
| Holmes et al. 2008 | WFD | Folate (µg) | 32 | M  (11-17y) | Food checklist | Self | 16 | -268 to 300 |
| Holmes et al. 2008 | WFD | Folate (µg) | 16 | F  (11-17y) | Food checklist | Self | 21 | -115 to 157 |
| Cade et al. 2006 | WFD | Folate (µg) | 180 | M&F  (3-7y) | Food checklist | Parent/Interviewer/ self | 23 | -118 to 164 |
| Holmes et al. 2008 | WFD | Vitamin C (mg) | 19 | M  (11-17y) | S-WFD | Self | -7 | -147 to 133 |
| Holmes et al. 2008 | WFD | Vitamin C (mg) | 11 | F  (11-17y) | S-WFD | Self | -31 | -103 to 41 |
| Holmes et al. 2008 | WFD | Vitamin C (mg) | 21 | M  (2-10y) | S-WFD | Parent | 6 | -74 to 86 |
| Holmes et al. 2008 | WFD | Vitamin C (mg) | 23 | F  (2-10y) | S-WFD | Parent | 7 | -131 to 145 |
| Holmes et al. 2008 | WFD | Vitamin C (mg) | 38 | M  (2-10y) | MPR | Parent | 16 | -76 to 108 |
| Holmes et al. 2008 | WFD | Vitamin C (mg) | 38 | F  (2-10y) | MPR | Parent | 28 | -98 to 154 |
| Holmes et al. 2008 | WFD | Vitamin C (mg) | 16 | F  (11-17) | MPR | Self | 15 | -75 to 105 |
| Holmes et al. 2008 | WFD | Vitamin C (mg) | 32 | M  (11-17y) | MPR | Self | 4 | -108 to 116 |
| Holmes et al. 2008 | WFD | Vitamin C (mg) | 38 | M  (2-10y) | Food checklist | Parent | 33 | -87 to 153 |
| Holmes et al. 2008 | WFD | Vitamin C (mg) | 38 | F  (2-10y) | Food checklist | Parent | 43 | -101 to 187 |
| Holmes et al. 2008 | WFD | Vitamin C (mg) | 32 | M  (11-17y) | Food checklist | Self | 18 | -150 to 186 |
| Holmes et al. 2008 | WFD | Vitamin C (mg) | 16 | F  (11-17y) | Food checklist | Self | 24 | -168 to 216 |
| Cade et al. 2006 | WFD | Vitamin C (mg) | 180 | M&F  (3-7y) | Food checklist | Parent/Interviewer/ self | 6.5 | -138 to 150 |
| Christian et al. 2015  (Cade) | WFD | Vitamin C (mg) | 67 | M&F  (8-11y) | Food checklist | Parent/Interviewer/ self | -27 (median) | No results |
| Holmes et al. 2008 | WFD | Calcium (mg) | 21 | M  (2-10y) | S-WFD | Parent | -77 | -663 to 509 |
| Holmes et al. 2008 | WFD | Calcium (mg) | 23 | F  (2-10y) | S-WFD | Parent | 38 | -438 to 514 |
| Holmes et al. 2008 | WFD | Calcium (mg) | 19 | M  (11-17y) | WFD | Self | 48 | -534 to 630 |
| Holmes et al. 2008 | WFD | Calcium (mg) | 11 | F  (11-17y) | WFD | Self | 43 | -509 to 595 |
| Holmes et al. 2008 | WFD | Calcium (mg) | 38 | M  (2-10y) | MPR | Parent | 15 | -565 to 595 |
| Holmes et al. 2008 | WFD | Calcium (mg) | 38 | F  (2-10y) | MPR | Parent | 133 | -329 to 595 |
| Holmes et al. 2008 | WFD | Calcium (mg) | 16 | F  (11-17y) | MPR | Self | 39 | -437 to 515 |
| Holmes et al. 2008 | WFD | Calcium (mg) | 32 | M  (11-17y) | MPR | Self | 142 | -460 to 744 |
| Holmes et al. 2008 | WFD | Calcium (mg) | 38 | M  (2-10y) | Food checklist | Parent | 60 | -568 to 699 |
| Holmes et al. 2008 | WFD | Calcium (mg) | 38 | F  (2-10y) | Food checklist | Parent | 89 | -291 to 469 |
| Holmes et al. 2008 | WFD | Calcium (mg) | 32 | M  (11-17y) | Food checklist | Self | 39 | -583 to 661 |
| Holmes et al. 2008 | WFD | Calcium (mg) | 16 | F  (11-17y) | Food checklist | Self | 77 | -385 to 539 |
| Cade et al. 2006 | WFD | Calcium (mg) | 180 | M&F  (3-7y) | Food checklist | Parent/Interviewer/ self | 9 | -673 to 691 |
| Christian et al. 2015  (Cade) | WFD | Calcium (mg) | 67 | M&F  (8-11) | Food checklist | Parent/Interviewer/ self | 185 | -466 to 836 |
| Lietz et al. 2002  (McKeown) | WFD | Calcium (mg) | 50 | M&F  (11-13y) | FFQ | Self | 203 | -393 to 799 |
| Christian et al. 2015  (Cade) | WFD | Sodium (mg) | 67 | M&F  (8-11y) | Food checklist | Parent/Interviewer/ self | 151 | -2879 to 3181 |
| Albar et al. 2016  (Carter) | Recall | Energy (kcal) | 75 | M&F  (11-18y) | MPR | Self | -55 | -797 to 687 |
| Albar et al. 2016  (Carter) | Recall | Protein (g) | 75 | M&F  (11-18y) | MPR | Self | -2 | -45 to 41 |
| Albar et al. 2016  (Carter) | Recall | Fat (g) | 75 | M&F  (11-18y) | MPR | Self | -3 | -52 to 46 |
| Albar et al. 2016  (Carter) | Recall | CHO (g) | 75 | M&F  (11-18y) | MPR | Self | -11 | -152 to 130 |

WFD= Weighed Food Diary; S-WFD= Semi-Weighed Food Diary; EFD= Estimated Food Diary; MPR= Multiple pass recall; FFQ= Food Frequency Questionnaire; M&F=Male and Female
